# Comparing the application of three thrombosis risk assessment models in patients with acute poisoning: A cross-sectional survey

**DOI:** 10.3389/fmed.2022.1072467

**Published:** 2022-12-02

**Authors:** Zixin Wen, Xiuqin Li, Yanxia Zhang, Jie Shi, Juan Zhang, Yingying Zheng, Ying Lin, Tianzi Jian, Xiangdong Jian, Baotian Kan, Xiaorong Luan

**Affiliations:** ^1^School of Nursing and Rehabilitation, Cheeloo College of Medicine, Shandong University, Jinan, China; ^2^Department of Geriatric Medicine, Qilu Hospital, Cheeloo College of Medicine, Shandong University, Jinan, China; ^3^Nursing Theory and Practice Innovation Research Center, Shandong University, Jinan, China; ^4^Department of Poisoning and Occupational Diseases, Emergency Medicine, Qilu Hospital, Cheeloo College of Medicine, Shandong University, Jinan, China; ^5^School of Public Health, Cheeloo College of Medicine, Shandong University, Jinan, China; ^6^Department of Nursing, Qilu Hospital, Cheeloo College of Medicine, Shandong University, Jinan, China

**Keywords:** acute poisoning, hemoperfusion, deep venous thrombosis, thrombosis risk assessment, predictive value

## Abstract

**Background:**

Patients with acute toxic hemoperfusion are prone to deep vein thrombosis. However, there is no risk assessment model for thrombosis in patients with acute toxic hemoperfusion. Therefore, we compared three commonly used risk assessment models for deep vein thrombosis to determine the model most suitable for assessment of deep vein thrombosis in patients with acute toxic hemoperfusion.

**Methods:**

Caprini, Autar, and Padua thrombosis risk assessment models were used to assess the risk of deep vein thrombosis in patients with acute poisoning and hemoperfusion admitted to a grade A hospital in Shandong province from October 2017 to February 2019. The predictive values of the three models were compared using receiver operating characteristic (ROC) curve analysis.

**Results:**

The risk assessment model scores of Caprini, Autar, and Padua were 7.55 ± 1.76, 8.63 ± 2.36, and 3.92 ± 0.55, respectively. The Caprini risk assessment model was significantly different (*p* < 0.05) in high-risk patients in the thrombus and non-thrombotic groups; the difference between the other two models was not significant (*p* > 0.05). The areas under the ROC curve of the Caprini, Autar, and Padua risk assessment models were 0.673, 0.585, and 0.535, respectively. The difference in areas under the ROC curve between the Caprini risk assessment model and the Autar risk assessment model as well as the Padua risk assessment model was significant (*p* < 0.05), but the areas under the ROC curve of the Autar risk assessment model and the Padua risk assessment model were not statistically significant (*p* > 0.05). The Caprini risk assessment model had a sensitivity of 91.9%, specificity of 33.1%, and a Youden index of 0.249. The sensitivity and specificity of Autar’s risk assessment model were 37.0 and 77.2%, respectively, and the Youden index was 0.141. The Padua risk assessment model had a sensitivity of 91.3%, specificity of 15.0%, and a Youden index of 0.063.

**Conclusion:**

The three thrombosis risk assessment models were not suitable for patients with acute poisoning and hemoperfusion.

## Introduction

In China, poisoning and injury are the fifth leading cause of death among residents (1). Acute poisoning refers to a series of pathophysiological changes and clinical manifestations that occur after the human body is exposed to toxic substances or high doses of toxic drugs within a short period of time. Patients with acute poisoning are critically ill, and most poisons lack specific antidotes. Hemoperfusion (HP) can quickly remove poisons and metabolites in patients and effectively eliminate excessive inflammatory mediators in the blood. Moreover, it has the advantages of simple operation, short treatment time, and low total cost and has become an important means of rescue from various types of acute poisoning (2). An indwelling central venous catheter should be set up in patients undergoing hemoperfusion, while the femoral vein catheter is widely used in patients with acute poisoning because of its simplicity and high success rate. Thrombosis after catheterization is the most common catheter-related complication in patients undergoing hemodialysis with femoral vein indwelling catheters. Once it occurs, catheterization can only be performed by extubation and site replacement, which increases patients’ medical costs and pain.

Since the beginning of the 21st century, thrombotic diseases have accounted for 25% of the global mortality rate (3). Among them, venous thromboembolism is the third most common vascular disease after acute coronary syndrome and stroke. It is also the main preventable cause of death in hospitals, including deep vein thrombosis (DVT) and pulmonary embolism (4, 5). DVT is an obstacle to venous return caused by abnormal blood clotting in the deep veins and usually occurs in the lower limbs. The shedding of thrombus from the deep vein system and blockage of the pulmonary artery or its branches leads to a pulmonary circulation disorder called pulmonary embolism (6). Once pulmonary embolism occurs, the patient’s life is seriously endangered. Therefore, it is important to accurately assess the risk of DVT in patients with acute poisoning who undergo hemoperfusion to correctly implement preventive measures, since they are a high-risk group for DVT.

Because the clinical symptoms of DVT of the lower limbs are atypical and critically ill patients often have altered consciousness that hinders the acquisition of disease-related information, the condition is often hidden, and clinicians often use imaging (color Doppler ultrasound, venography, magnetic resonance venous imaging, and thromboelastic mapping) and laboratory indicators to diagnose DVT. Although the above examinations have high accuracy, they are expensive and time-consuming, and an effective, convenient, and economic risk prediction tool for DVT occurrence is urgently needed. Currently, the commonly used thrombosis risk assessment models include Wells, Caprini, Autar, and Padua (7–10), whose risk factors and score settings are different; their predictive values are also different. However, there is no clear regulation regarding which thrombosis risk assessment model should be chosen for patients undergoing hemoperfusion with acute poisoning. Therefore, this study compared the predictive values of Caprini, Autar, and Padua, commonly used thrombosis risk assessment models, among patients undergoing hemoperfusion with acute poisoning to identify a more suitable thrombosis risk assessment tool.

## Materials and methods

### Study population

Convenience sampling was used to select patients with acute poisoning and hemoperfusion who visited the poisoning department of a grade A hospital in Shandong province from October 2017 to February 2019. The sample size calculation in this study was equivalent to that of the diagnostic test, and the formula was as follows: n = (Zα/δ) ^2^/P (1–P). When the test level α was set at 0.05, Zα = Zα/2 = 1.96, and δ was the cut-off value, generally 0.05–0.10. In this study, δ was set at 0.10, and P was the sensitivity or specificity. Sensitivity and specificity values are typically used to estimate the sample size required for case and control groups. According to previous literature and statistical theory, a model with better predictive ability should have higher sensitivity and specificity. Sensitivity determines the sample size required for the thrombus group, while specificity determines the sample size required for the non-thrombus group. The sensitivity and specificity in this study were set at 80 and 75%, respectively. The sample size of the thrombus group should be approximately 62, and that of the non-thrombus group, approximately 72. The inclusion criteria were as follows: (1) acute poisoning and (2) temporary femoral vein catheterization and hemoperfusion therapy. Exclusion criteria were: (1) taking drugs and poisons that affect clotting function (such as aspirin and warfarin or anticoagulant rodenticide poisoning); (2) having underlying diseases that affect the coagulation and/or fibrinolytic system (such as primary blood system diseases and platelet-related diseases); and (3) having incomplete medical records. In total, 311 patients with acute poisoning and hemoperfusion were included in this study. The participants were divided into a thrombus group (*n* = 184) and a non-thrombus group (*n* = 127) according to the results of ultrasound examination before extubation.

### Research methods

After the patient’s condition stabilized on the day of admission, general information was collected, including the patient’s age, sex, type of poisoning, and past medical history. The Caprini (11, 12), Autar (13), and Padua (14) risk assessment models (as presented in the Supplementary material) were used to score and grade the risk of DVT in patients with acute hemoperfusion poisoning on a daily basis until the patient was extubated. The highest score of each risk assessment model was included in this study. This study was approved by the Ethics Committee of Qilu Hospital of Shandong University in China (No. KYLL-2018-163), and we obtained informed consent from the patients. The study was carried out in accordance with the Declaration of Helsinki and related guidelines/provisions.

### Statistical analysis

An Excel spreadsheet was created to sort the data, and two investigators populated the table. SPSS 22.0 statistical software (IBM Corp., Armonk, NY, USA) was used for statistical analysis. Measurement data consistent with normal distribution were descriptively analyzed and presented as means and standard deviations, and the difference was analyzed using independent-sample *T*-test or analysis of variance. The median and quartile were used for the statistical description of measurement data that did not conform to the normal distribution, and the Mann–Whitney U test was used for difference analysis. The adoption rate and percentage of counting data are statistically described. The chi-square test was used for difference analysis. Statistical significance was set at a *p*-value of < 0.05. SPSS 22.0 software was used to draw the receiver operating characteristic (ROC) curve of the thrombosis risk assessment model, and the area under the ROC curve was calculated. MedCalc software was used to compare the area under the ROC curve (AUC), sensitivity, and specificity of the three thrombosis risk assessment models.

## Results

### Demographic characteristics of patients

In total, 311 patients with acute poisoning were included in this study [64 women (52.7%); 147 men (47.3%), and average age of 34.73 ± 15.93 years] (Table 1).

**TABLE 1:** Patient demographic characteristics.

Project	Group	Frequency	Constituent
			ratio (%)
Sex	Male	164	52.73
	Female	147	47.27
Poisoning category	Paraquat	194	62.38
	Organophosphorus	31	9.96
	Aquacide	23	7.40
	Other herbicides	23	7.40
	Other insecticides	12	3.86
	Other	28	9.00
Body Mass Index	< 18.5 kg/m^2^	36	11.58
	18.5–22.9 kg/m^2^	139	44.69
	23.0–24.9 kg/m^2^	68	21.86
	25.0–29.9 kg/m^2^	51	16.40
	> 30 kg/m^2^	17	5.47
Type of comorbidity	Hypertension	21	6.75
	Diabetes	8	2.57
	Coronary heart disease	3	0.96
	Cerebrovascular disease	5	1.61
Ultrasound examination for VTE	Yes	184	59.16
	No	127	40.84

VTE, venous thrombo-embolism.

### Comparison of the thrombosis risk scores of patients with acute poisoning using the three thrombosis risk assessment models

When the thrombosis risk of patients with acute poisoning was assessed using the Caprini and Autar risk assessment models, the scores of the thrombus and non-thrombus groups were significantly different (*P* < 0.05). There was no significant difference between the two groups using the Padua risk assessment model (*P* > 0.05). In terms of screening high-risk patients, the Caprini risk assessment model showed a significant difference compared to the other two groups (*P* > 0.05), while the Autar and Padua risk assessment models showed no significant difference compared to the other two groups (*P* < 0.05), as shown in Table 2.

**TABLE 2:** Comparison of VTE risk scores in patients with poisoning using the three thrombosis risk assessment models.

Group	Caprini risk assessment model		Autar risk assessment model		Padua risk assessment model	
	Score [M(P25,P75)]	High-risk patients [*n*(%)]	Score [M(P25,P75)]	High-risk patients [*n*(%)]	Score [M(P25,P75)]	High-risk patients [*n*(%)]
Thrombus group	8 (8∼9)	181(98.4)	9 (7∼10)	6(3.3)	4 (4∼4)	169(91.8)
Non-thrombus group	8 (5∼8)	118(92.9)	8 (7∼9)	2(1.6)	4 (4∼4)	108(85.0)
Z/χ^2^	–5.351	4.649	–2.577	0.312	–1.585	3.577
*P*	<0.001	0.031	0.010	0.576	0.113	0.059

### Comparison of area under the receiver operating characteristic curves for predicting deep venous thrombosis in patients with acute poisoning using the three thrombotic risk assessment models

**FIGURE 1:**
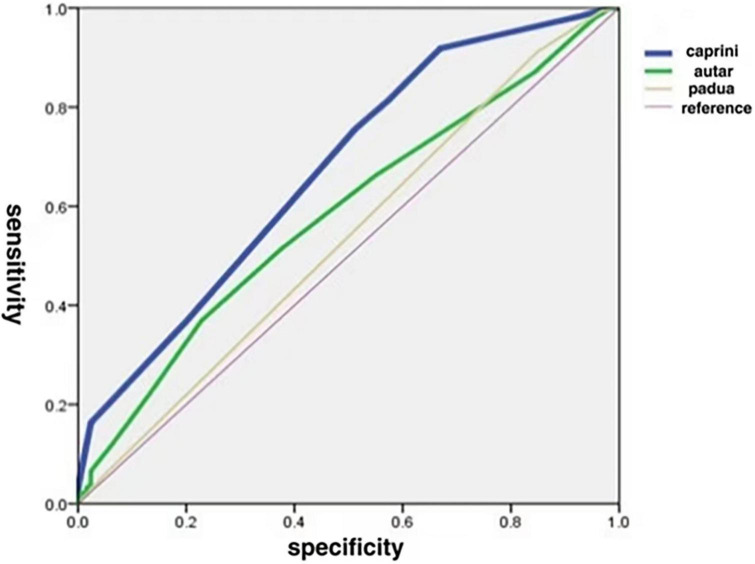
Comparison of areas under ROC curve for the three thrombosis risk assessment models. ROC, receiver operating characteristics.

The AUCs of the Caprini, Autar, and Padua risk assessment models are 0.673 ± 0.030, 0.585 ± 0.032, and 0.535 ± 0.023, respectively (Figure 1 and Table 3). Comparison of the AUCs of the three models using MedCalc software shows that the AUC values of the Caprini risk assessment model and Autar risk assessment model, as well as the Caprini risk assessment model and Padua risk assessment model, were significantly different (*P* < 0.05). The AUC values of the Autar and Padua risk assessment models showed no significant difference (*P* > 0.05; Table 4).

**TABLE 3:** Sensitivity, specificity, Youden index, and AUC of the three risk assessment models for thrombosis.

	Sensitivity (%)	Specificity (%)	Youden index	Positive likelihood ratio	Negative likelihood ratio	AUC	95% CI
Caprini Model	91.9	33.1	0.249	1.37	0.25	0.673 ± 0.030	0.618∼0.725
Autar Model	37.0	77.2	0.141	1.62	0.82	0.585 ± 0.032	0.528∼0.640
Padua Model	91.3	15.0	0.063	1.07	0.58	0.535 ± 0.023	0.478∼0.591

AUC, area under the ROC curve; CI, confidence interval.

**TABLE 4:** Comparison of AUCs for the three thrombosis risk assessment models.

	Risk assessment model		Risk assessment model		Risk assessment model	
	Caprini	Autar	Caprini	Padua	Autar	Padua
AUC	0.673	0.585	0.673	0.535	0.585	0.535
z statistic	2.727		4.517		1.649	
*P*	0.006		<0.001		0.099	

AUC, area under the ROC curve.

## Discussion

In this study, the number of thromboses reported accounted for 59.2% of the total, which is higher than the 8–40% incidence of DVT reported in the intensive care unit (15). This may be because all the included patients underwent femoral vein catheterization, and catheter-related thrombosis is a common complication after central vein catheterization. This is related to multiple factors such as puncture damage to the vein wall and slow blood flow after catheterization (16). After catheterization, the integrity of the blood vessels is damaged, and vascular endothelial cells produce procoagulant factors that activate platelets and promote blood coagulation, leading to the formation of an early thrombus. Furthermore, prolonged catheter placement in the vein causes mechanical stimulation of the vascular epithelial cells and vascular endothelial damage. As a foreign body, an indwelling catheter is not conducive to blood return, causing local vascular inflammation and leading to thrombi that are easily formed. Catheter placement makes the vascular lumen relatively narrow, resulting in relatively slow local blood flow and thrombosis. Therefore, for these patients, nursing staff should repeatedly evaluate the duration of use and patency of the femoral vein catheter and replace the catheters timely after prolong use or when dislodged.

Currently, the commonly used clinical thrombosis risk assessment models include Wells, Caprini, Autar, and Padua. However, studies have pointed out that the Wells model is more suitable for outpatients (17); therefore, we selected the Caprini, Autar, and Padua risk assessment models for thrombosis. In this study, the Caprini risk assessment model was superior to the Autar and Padua models in assessing DVT risk in patients with acute toxic hemoperfusion. By comparing the thrombus risk scores of patients in the thrombus and non-thrombus groups, the scores of the Caprini and Autar risk assessment models were observed to be significantly different compared to the other two groups (*P* < 0.05). Additionally, the scores of the thrombus group were higher than those of the non-thrombus group, while the Padua risk assessment model showed no significant difference compared to the other two groups (*P* > 0.05). These results indicate that the Caprini and Autar risk assessment models can effectively assess DVT risk in patients undergoing hemoperfusion after acute poisoning. In terms of screening high-risk patients, 98.4% of patients with acute poisoning caused by DVT were screened using the Caprini model, while only 3.3% of high-risk patients were screened using the Autar model, indicating that the Caprini model has a higher sensitivity in screening high-risk patients. This may be due to the inclusion of multiple orthopedic factors in the Autar model, such as pelvic and lower limb trauma, which are highly targeted for patients undergoing orthopedic surgery (9).

In this study, the AUCs of the Caprini, Autar, and Padua risk assessment models were 0.673 ± 0.030, 0.585 ± 0.032, and 0.535 ± 0.023, respectively, and the AUC was between 0.5–0.7. The Caprini risk assessment model had the largest AUC, which was significantly different from that of the other two models (*P* < 0.05). Both Caprini and Padua risk assessment models have high sensitivity, but the Padua is not as specific as the Caprini model. Although the specificity of the Autar risk assessment model was higher than that of the Caprini model, its sensitivity was much lower. These results suggest that these three risk assessment models are not suitable for predicting the occurrence of DVT in patients with acute toxic hemoperfusion. This may be related to the differences in physiological characteristics, eating habits, and mutation rates of thrombo-related pathogenic genes between the European, American, and Chinese populations, which are the main research objects of the proposed Caprini, Autar, and Padua models. Moreover, due to their critical condition, patients with acute poisoning often show coagulation system activation, prothrombin reduction, fiber system activation, and inhibition of the two-way reaction phenomenon. However, during hemoperfusion, the perfusion device can absorb platelets, coagulation factors, and fibronectin in the blood (18), whereas anticoagulation with heparin sodium can inhibit the activity of coagulation factors and prevent platelet aggregation and activation (19), making patients more prone to abnormal coagulation function, leading to low accuracy of the prediction model.

There are some shortcomings in this study. Firstly, only patients with femoral vein catheterization were included in this study, and the effect of catheterization site on thrombosis risk was not considered. Secondly, this is a single-center study, and a larger-sample multicenter study can be conducted in the future. Despite some shortcomings, this study suggests the lack of an appropriate predictive model for DVT in patients with acute poisoning and hemoperfusion.

This study is a preliminary study to assess the risk of thrombosis in patients with acute poisoning and hemoperfusion. Based on the results of this study, we constructed a thrombosis risk model in patients with acute poisoning and hemoperfusion, which has recently been published (20).

## Conclusion

The three thrombosis risk assessment models are not applicable to patients with acute poisoning and hemoperfusion. In future research, a prediction model for DVT in patients with acute toxic hemoperfusion needs to be explored, and reasonable preventive measures can be formulated according to its influencing factors.

## Data availability statement

The raw data supporting the conclusions of this article will be made available by the authors, without undue reservation.

## Ethics statement

The studies involving human participants were reviewed and approved by the Ethics Committee of Qilu Hospital of Shandong University in China. The patients/participants provided their written informed consent to participate in this study.

## Author contributions

ZW and XQL conceived and designed the study and wrote the manuscript. YaZ, JS, JZ, YiZ, YL, and TJ collected and analyzed the data. BK, XRL, and XJ reviewed and edited the manuscript. All authors read and approved the manuscript and agreed to be accountable for all aspects of the research in ensuring that the accuracy or integrity of any part of the work are appropriately investigated and resolved.

## Abbreviations

HP: hemoperfusion
DVT: deep venous thrombosis
AUC: area under the ROC curve
ROC: receiver operating characteristic.
