# Supplementary material for: Comparing the application of three thrombosis risk assessment models in patients with acute poisoning: A cross-sectional survey
Source: Front Med (Lausanne). 2022 Dec 2;9:1072467. doi: 10.3389/fmed.2022.1072467 (PMC9755164; doi:10.3389/fmed.2022.1072467)
Supplement: Supplementary file 1 [file Presentation_1.pdf]

# *Supplementary Material*

## 1 Risk assessment models

### 1.1 Caprini Risk Assessment model for thrombosis

The Caprini Risk Assessment was first designed by Caprini, an American scholar, and colleagues in 1991 (1). In 2012, the American Association of Chest Physicians Clinical Practice Guidelines for Antithrombotic Therapy and Thrombosis Prevention in Non-orthopedic Surgery Patients (9th edition) recommended the Caprini risk assessment tool as a risk screening tool for VTE in patients undergoing non-orthopedic surgery (2). The risk evaluation model included a total of approximately 40 factors such as age, obesity (body mass index [BMI]) of 25 kg/m2, bedridden, at high risk of disease, surgery, trauma, and laboratory examination; points were assigned to each risk factor. Patients with risk can be divided into low risk (0-2 points), moderate risk (3-4 points), and high-risk (≥5 points) or higher categories according to the total score obtained.

### 1.2 Autar risk assessment model for thrombosis

The Autar risk assessment was developed by Autar, a British nursing expert, in 1996, based on the three factors of venous thrombosis and is currently most commonly used to assess the risk of thrombosis in patients undergoing trauma and orthopedic surgery (3,4). The risk assessment model included seven dimensions, including age, BMI, activity level, trauma risk, special risk, high-risk disease, and surgery. Each risk factor was assigned a score of 1-7, with overall scores of 0-10 (low risk), 11-14 (medium risk), and ≥15 (high risk).

### 1.3 Padua thrombosis risk Assessment model

The Padua thrombosis risk assessment was designed and developed by Italian scholars Barbar et al. in 2010 and is mostly used for inpatients in internal medicine (5). The risk evaluation model included age ≥70 years, braking, obesity (BMI ≥30 kg/m2), malignant tumor, active, history of venous thrombosis, thrombophilia or antithrombin deficiency, heart failure and/or respiratory failure, acute myocardial infarction and/or cerebral ischemic stroke within a month, trauma or surgery, acute infection and/or rheumatoid disease, and hormone replacement therapy. Each risk factor was assigned a score of 1-3, with an overall score of 0-3 being low risk and ≥4 being high risk.

**Reference**


1. Caprini JA. Risk assessment as a guide for the prevention of the many faces of venous thromboembolism. Am J Surg (2010) 199:S3-S10. doi:10.1016/j.amjsurg.2009.10.006.

2. Gould MK, Garcia DA, Wren SM, Karanicolas PJ, Arcelus JI, Heit JA, et al. Prevention of VTE in nonorthopedic surgical patients: antithrombotic therapy and prevention of thrombosis, 9th ed: American College of Chest Physicians evidence-based clinical practice guidelines. Chest, 9th ed (2012) 141:e227S-77S. doi:10.1378/chest.11-2297.

3. Büyükyılmaz F, Şendir M, Autar R, Yazgan İ. Risk level analysis for deep vein thrombosis (DVT): A study of Turkish patients undergoing major orthopedic surgery. J Vasc Nurs (2015) 33:100-5. doi:10.1016/j.jvn.2015.01.004.

4. Autar R. Nursing assessment of clients at risk of deep vein thrombosis (DVT): The Autar DVT scale. J Adv Nurs (1996) 23:763-70. doi:10.1111/j.1365-2648.1996.tb00049.x.

5. Barbar S, Noventa F, Rossetto V, Ferrari A, Brandolin B, Perlati M, et al. A risk assessment model for the identification of hospitalized medical patients at risk for venous thromboembolism: The Padua Prediction Score. J Thromb Haemost (2010) 8:2450-7. doi:10.1111/j.1538-7836.2010.04044.x.
